# Effect of lipopolysaccharide and polyinosinic:polycytidylic acid in a murine model of nasal polyp

**DOI:** 10.1038/s41598-020-80483-y

**Published:** 2021-01-13

**Authors:** Jee Hye Wee, Young-Kyung Ko, Roza Khalmuratova, Hyun-Woo Shin, Dae Woo Kim, Chae-Seo Rhee

**Affiliations:** 1 Department of Otorhinolaryngology-Head and Neck Surgery, Hallym University Sacred Heart Hospital, Hallym University College of Medicine, Anyang, Korea; 2 Graduate School of Medicine, Seoul National University College of Medicine, Seoul, Korea; 3 Department of Pharmacology, Ischemic/Hypoxic Disease Institute, Seoul National University College of Medicine, Seoul, Korea; 4 Department of Otorhinolaryngology-Head and Neck Surgery, Seoul National University Hospital, Seoul National University College of Medicine, 101 Daehagro, Jongro-gu, Seoul, 03080 Korea; 5 Department of Otorhinolaryngology-Head and Neck Surgery, Boramae Medical Center, Seoul National University College of Medicine, Seoul, Korea

**Keywords:** Inflammation, Mucosal immunology, Immunological disorders, Respiratory tract diseases

## Abstract

Several factors, including bacterial and viral infections, have been associated with rhinosinusitis and nasal tissue remodelling that may result in nasal polyp formation. However, the potential role of bacterial or viral stimuli triggering polyp development is unclear. Here, we used lipopolysaccharide (LPS) and polyinosinic:polycytidylic acid [poly(I:C)] in a murine model of allergic rhinosinusitis to compare different effects of bacterial- and virus-derived stimuli in the pathogenesis of nasal polyp formation. Briefly, BALB/c mice were sensitised and challenged with ovalbumin and staphylococcal enterotoxin, with or without LPS or poly(I:C), and the consequent histopathological profiles, cytokines, and systemic humoral responses were studied. While no significant differences in polyp formations and epithelial disruptions were observed among the experimental groups, the local cell recruitment patterns slightly differed in animals that received either LPS or poly(I:C). Additionally, the local immune environments generated by LPS or poly(I:C) stimulation varied. LPS stimulation induced a marked Th1/Th17 response and predominantly neutrophilic nasal polyp formations, whereas poly(I:C) induced a Th2-skewed environment in neutrophilic nasal polyp development. Overall, our findings show that both cell recruitment patterns and local immune environments induced by these two stimuli differ, which may have implications in the physiopathology of rhinosinusitis with nasal polyp.

## Introduction

Chronic rhinosinusitis with nasal polyps (CRSwNP) is a common chronic inflammatory disease of the paranasal sinuses. There are different phenotypes of nasal polyps (NPs), dependent on the type of immune cell infiltration and local cytokine environment. In Western patients, NPs are characterised as secondary to Th2-dominant immune responses, including the predominance of eosinophil infiltration together with the excessive expression of type 2 cytokines^1^. In contrast, in Asian patients, neutrophilic NPs, associated with Th1 and Th17 responses are typically observed^2,3^. These differences between Western and Asian patients suggest that NP endotypes differ according to the patient ethnicity. However, NP formation results from a combination of individual susceptibility and environmental factors. In fact, a recent study suggests that a diversity of environmental factors, such as microbiota and air pollution, can influence Th cytokine profiles in patients with CRSwNP^4^. Furthermore, a new conception proposed that the occurrence of mechanical dysfunction within the nasal mucosa tissues promotes NP development^5,6^.

Several studies suggest that *Staphylococcus aureus* colonization and the resultant enterotoxin-specific IgE secretion is associated with the pathogenesis of NP formation and eosinophilic inflammation, which is in line with the superantigen hypothesis^7–9^. Therefore, we previously established the first murine model of eosinophilic NPs using ovalbumin (OVA) combined with staphylococcal enterotoxin B (SEB)^10^. We demonstrated that the NP phenotype depends on the SEB dosage: a higher SEB dose induces greater neutrophilic infiltration together with a higher level of interferon gamma (IFN)-γ than that by a lower-dose^10^. Furthermore, another study also reported that a high SEB dose induces interleukin (IL)-17A expression in mice^11^.

Lipopolysaccharide (LPS) is a fundamental constitutive block of the gram-negative bacterial cell wall and a potent activator of the immune system via Toll-like receptor (TLR) 4 recognition and signalling^12^. LPS is known to induce a strong airway inflammation and promote local neutrophil recruitment^13–15^. A few studies have already reported LPS-mediated neutrophil induction using murine models^16,17^. In fact, a neutrophil-dominant rhinitis model has been established with OVA and LPS intraperitoneal injections and showed that neutrophil recruitment is dependent on IL-17^16^. Additionally, a neutrophilic NP model has been developed with continuous intranasal instillations of LPS, that shows promotion of enhanced Th1- and Th17-related responses secondary to the TLR4 signalling pathways^17^.

Polyinosinic:polycytidylic acid [poly(I:C)] is a synthetic viral analogue^18^ used in experimental studies of immune responses to viral infections. These responses are initiated via TLR 3 signalling (after the recognition of single-stranded DNA molecules) that plays a critical role in the initiation of antiviral immunity. Previous studies showed that poly(I:C) triggers neutrophilic inflammation in asthma^19^ and rhinitis^20^ murine models.

Although several factors, including bacteria and viruses, have been associated with nasal tissue remodelling and rhinosinusitis^21–23^, the role of bacterial or viral stimuli in polyp development remains unclear. Therefore, the present study aimed to investigate whether LPS and poly(I:C), which are bacterial- and virus-derived stimuli, respectively, affect the pathogenesis of NP formation in a murine model of allergic rhinosinusitis with NP formation.

## Results

### Histology

**Figure 1:**
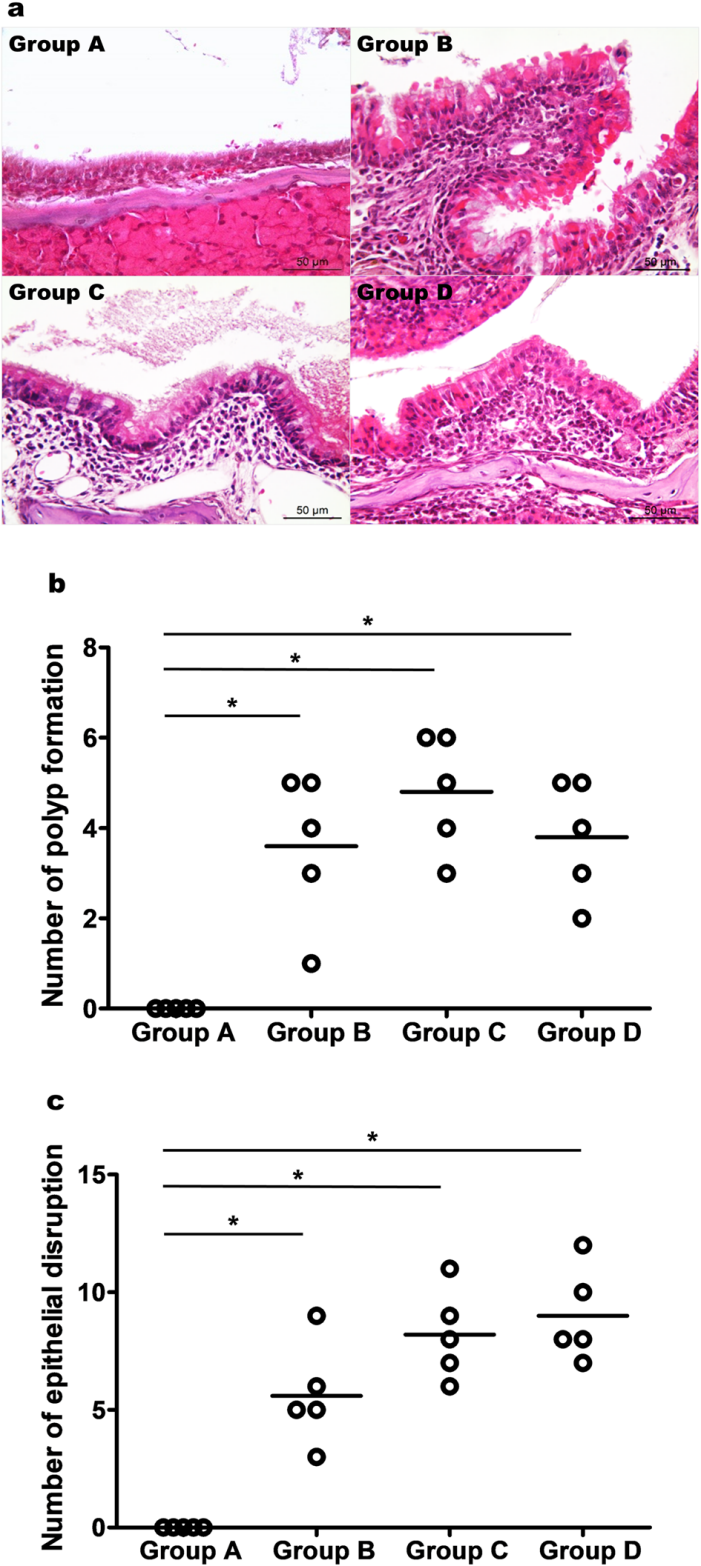
Histologic results of the development of polyps and epithelial disruptions. (**a**) Representative Hematoxylin & Eosin staining of the different experimental groups’ preparations. (**b**) Polyp formations and (**c**) epithelial disruptions. The data were plotted using GraphPad Prism version 5 (GraphPad Software Inc., San Diego, CA., USA, https://www.graphpad.com). Group A: negative control, Group B: positive control, Group C: LPS stimulation, Group D: poly(I:C) stimulation. *P < 0.05.

**Figure 2:**
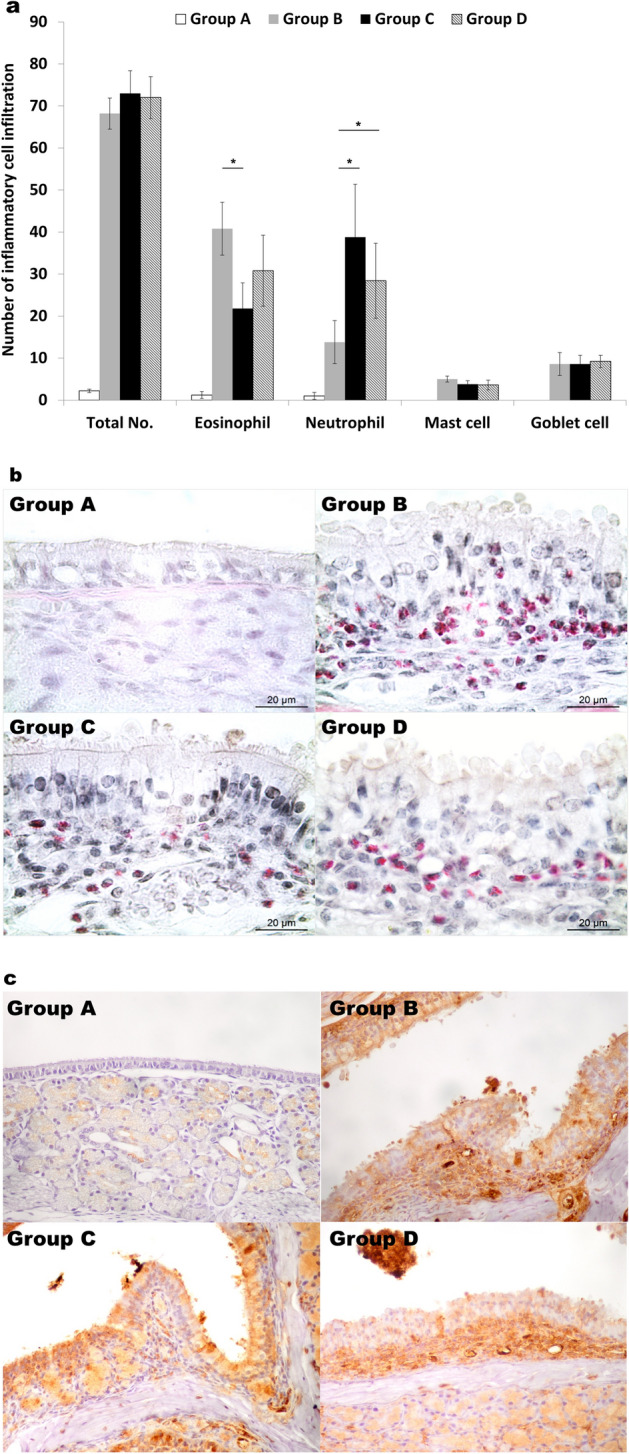
Histologic results of inflammatory cell infiltrations. (**a**) Inflammatory cell infiltrate profile of the different experimental groups. The data was plotted using Microsoft’s PowerPoint 2016 (https://www.microsoft.com). (**b**) Sirius red staining for eosinophils and (**c**) anti-neutrophil antibody staining. Group A: negative control, Group B: positive control, Group C: LPS stimulation, Group D: poly(I:C) stimulation. *P < 0.05.

To compare the different responses of bacterial- and virus-derived stimuli in NP development, we administered LPS and poly(I:C). As expected, the development of polyps and epithelial disruptions were observed in the positive control group (group B), compared with the negative control group (group A) (Fig. 1a). Additionally, these morphological alterations were also observed in both experimental groups (LPS: group C and poly(I:C): group D), to the same extent as that observed in the positive control group. Moreover, no significant differences in polyp formations (Fig. 1b; P = 0.469) and epithelial disruptions (Fig. 1c; P = 0.075) were detected among groups B, C, and D. Additionally, in groups B, C, and D, inflammatory cell infiltrates were observed to a greater extent than that in the negative control group (Fig. 2). With respect to cellularity, some differences were detected in the experimental and positive control groups. Animals that received LPS (group C) showed lower levels of eosinophil (Fig. 2a,b; P = 0.009) and higher levels of neutrophil (Fig. 2a,c; P = 0.009) infiltrations, respectively, compared with the positive control group (group B). Animals that received poly(I:C) (group D) showed higher levels of neutrophil infiltration than that in the positive control group (group B) (Fig. 2a,c; P = 0.027); however, although they showed increased eosinophil counts, the difference between groups B and D was not significant (Fig. 2a,b; P = 0.095). Mast (P = 0.071) and goblet cells (P = 0.804) infiltrations showed no differences between groups B, C, and D (Fig. 2a).

### Cytokines in the nasal mucosa

**Figure 3:**
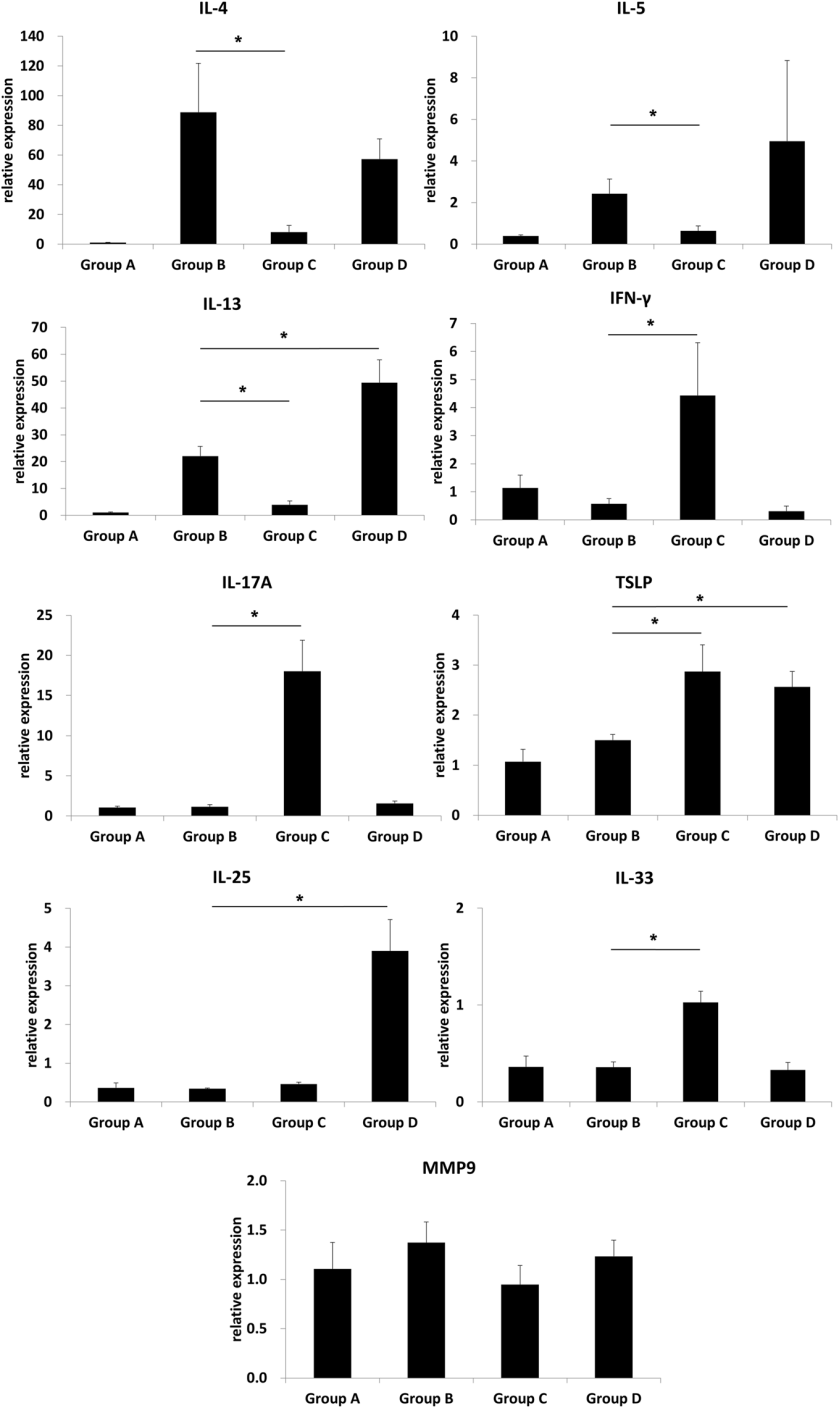
Comparison of the inflammatory cytokines profile of the nasal mucosa in different experimental groups. This figure was drawn using Microsoft’s PowerPoint 2016 (https://www.microsoft.com). Group A: negative control, Group B: positive control, Group C: LPS stimulation, Group D: poly(I:C) stimulation. *P < 0.05.

Tissue cellularity and immune tissue-environment are strictly related^24^. To understand if the abovementioned differences observed for infiltrating cells would translate to distinct tissue environments, we analysed expression of cytokines in the nasal mucosa. Figure 3 represents the overall cytokines in the nasal mucosae of different groups. The animals that received LPS as the co-sensitizer/co-challenging agent showed a significant upregulation of IFN-γ (P = 0.014) and IL-17A (P = 0.014) expression, along with significantly lower IL-4 (P = 0.016), IL-5 (P = 0.016), and IL-13 (P = 0.016) mRNA levels, compared with that in the positive control group. In contrast, the animals that received poly(I:C) showed a significantly increased IL-13 mRNA expression in the nasal mucosa (P = 0.047) compared with that in the positive control group. In the poly(I:C) group, the expressions of IL-4 (P = 0.016) and IL-5 (P = 0.047) were increased and that of IFN-γ (P = 0.101) and IL-17A (P = 0.101) were not different when compared with the negative control group; however, the mRNA expressions of IL-4 (P = 0.690), IL-5 (P = 0.421), IFN-γ (P = 0.730), and IL-17A (P = 0.175) showed no significant differences compared with that in the positive control group. Furthermore, while the TSLP mRNA levels were significantly increased in both LPS (P = 0.028) and poly(I:C) (P = 0.016) groups (compared with that in the positive control animals), IL-25 and IL-33 mRNA levels were significantly higher only in the poly(I:C) (P = 0.009; P = 0.754) or LPS (P = 0.117; P = 0.009) groups, respectively. Additionally, no changes in MMP9 gene expression were detected in the groups that received LPS (P = 0.286) or poly(I:C) (P = 0.486) compared with that in the positive control animals.

### Serum total and OVA-specific IgE levels

**Figure 4:**
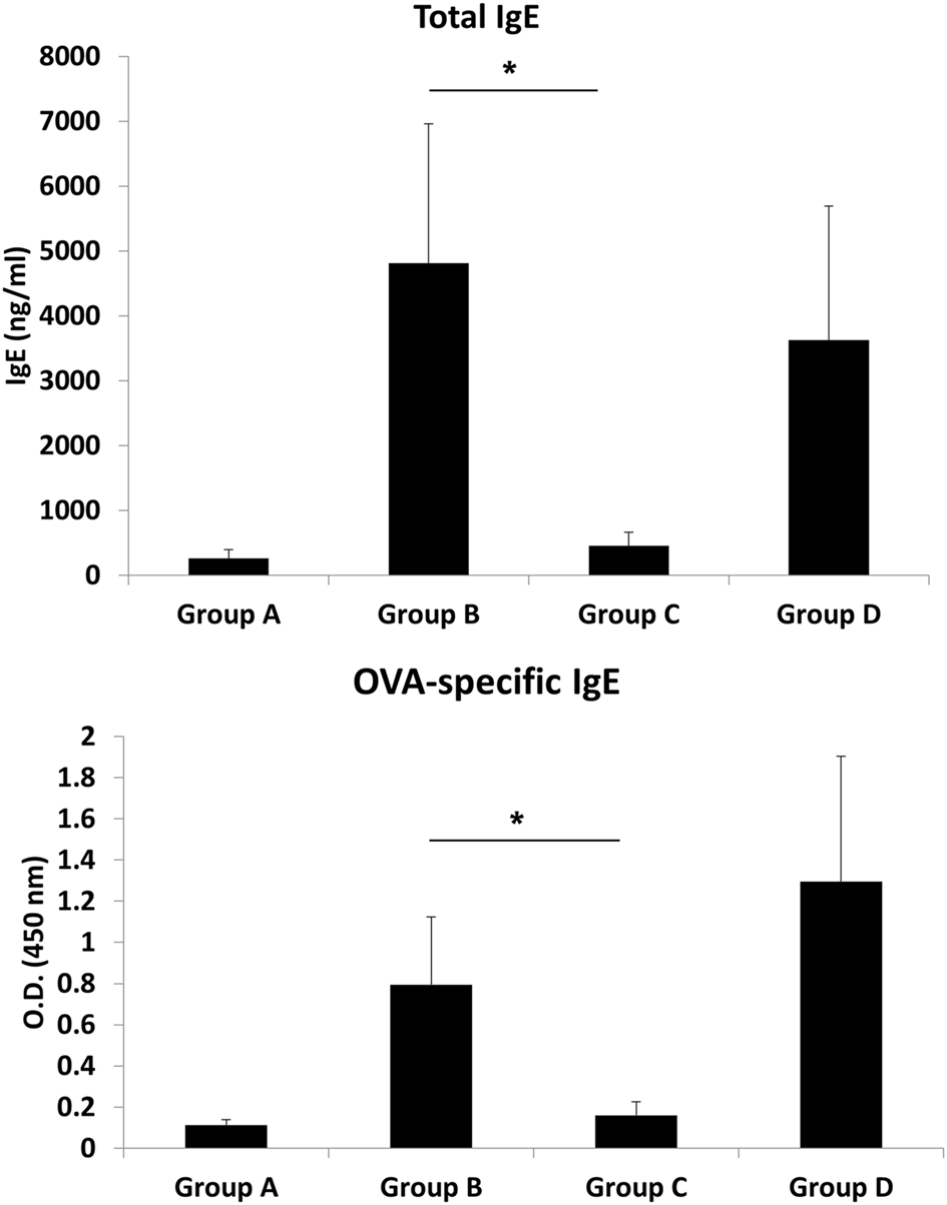
Serum total and OVA-specific IgE according levels. This figure was drawn using Microsoft’s PowerPoint 2016 (https://www.microsoft.com). Group A: negative control, Group B: positive control, Group C: LPS stimulation, Group D: poly(I:C) stimulation. *P < 0.05.

Allergy and IgE levels are intricately related^25^. As antibody production may be influenced by the immune environment^26^, we sought to understand if the reported differences in the nasal cavity cytokine expression would affect systemic IgE levels. The animals that received LPS showed significantly lower serum levels of total (P = 0.009) and OVA-specific (P = 0.014) IgE compared with that in the positive control group. Furthermore, in the animals receiving poly(I:C), the serum total (P = 0.056) and OVA-specific (P = 0.686) IgE levels were similar to that observed in positive control animals, however, the findings were not significant (Fig. 4).

## Discussion

Here, we used LPS and poly(I:C) in a murine model of allergic rhinosinusitis with NP to compare the different responses to bacterial- and virus-derived stimuli, respectively, in NP development. We found that these two stimuli induce different responses with respect to both cell recruitment patterns and local immune environments.

The co-administration of LPS as sensitizing/challenging agent predominantly induced neutrophilic infiltrates in the NP and Th1/Th17 immune environment. Our observation is consistent with previous studies reporting that LPS regulates the secretion of IL-17 in a variety of cell types through TLR4-mediated signalling^17,27,28^. Importantly, TLR4 has been shown to play critical roles in regulating the migration, activation, and life span of neutrophils^28,29^. Therefore, our data may provide further supporting evidence for the relationship between LPS-TLR4 crosstalk, IL-17 expression, and neutrophil infiltration.

Although the role of viral infections in CRSwNP development is not clear, herein we showed that the TLR3 agonist and viral analogue poly(I:C) promoted a Th2-skewed environment in neutrophilic nasal polyp development. Furthermore, data analysis hypothesizes that this is a consequence of the secretion of TSLP and IL-25. In fact, previous ex vivo studies reported that viral stimulation of polyp-derived epithelial cells enhanced the Th2 immune responses via the release of TSLP and IL-25 from epithelial cells^30,31^. However, in our in vivo study, the Th2-like nature was not as striking. This may be due to the model that was used. Administering a high SEB dose in an OVA-induced allergic chronic rhinosinusitis murine model was shown to induce high neutrophilic infiltration levels associated with increased expression of IFN-γ^10^. Although poly(I:C) stimulation in this context resulted in a distinct environment, we still observed a considerable amount of neutrophil infiltration and some-pro inflammatory cytokine secretion, which may indicate that our stimulatory conditions might not have completely counteracted the strong SEB-induced effect. Furthermore, in asthma^19^ and rhinitis^20^ animal models, contrary to the above-mentioned ex vivo models^29,30^), poly(I:C) was shown to promote significant mucosal neutrophil infiltration together with a mixed Th1/Th2 environment. In addition, there may be an effect of the dose of poly(I:C). Although in the present study, poly(I:C) was added at only 20 μg concentration in each of the experimental groups for the sensitization and challenge and a dose–response experiment was not performed, previous poly(I:C) induced asthma^19^ and rhinitis^20^ animal models have shown a dose-dependent neutrophilic inflammation.

In our study, IL-33 was upregulated by LPS, but not by poly(I:C) stimulation. IL-33 is thought to be the most probable triggering factor for Th2 immune responses in the mucosal tissues^32^. However, some studies also suggest that IL-33 has a role in neutrophil recruitment during infection^33,34^. In fact, a recent study reported that IL-33 plays a crucial role in the pathogenesis of neutrophilic inflammation in Asian patients with CRSwNP^35^. However, this may not be a universal fact, as another recent study has reported that IL‐33 expression is strongly influenced by geographically variable environmental factors^36^. Among these are infections caused by different agents. In line with this, and based on our data, we hypothesize that bacterial, but not viral, infections promote IL-33 secretion.

MMP9 is known to degrade collagen IV, which is the main component of the basement membrane that provides structural support to epithelial and endothelial cells^37^. Consequently, MMP9 secretion is thought to increase the microvasculature leakiness, promoting the transmigration of inflammatory cells and stromal oedema. Importantly, previous findings suggest that MMP9 is involved in the pathophysiology of NPs^38^. It has been shown that both TLR-4-^39^ and TLR3-^40^ mediated signalling promote MMP9 expression. However, in our study, no significant differences were detected in the MMP9 expression levels between animals that received LPS and poly(I:C) (TLR-4 and TLR-3 ligands, respectively) and the experimental positive controls. Again, this may be due to the choice of model. Importantly, the fact that we did not observe any significant differences in the NP formation between these three groups aligns with the comparable MMP9 expression levels determined.

The establishment of an appropriate murine model of CRSwNP is extremely important for the quest for new therapies to prevent NP formation. Wang et al. established a mouse model of LPS-induced neutrophilic NPs^17^, which reproduces the dominant Th1/Th17 responses observed in Asian patients^4^. NPs were found to induced by continuous intranasal instillation of LPS alone^17^. However, when we attempted a similar experimental approach, we did not observe any polyp formation at 15 weeks after continuous intranasal administration of 10, 20, or 50 μg of LPS (data not shown). This was the main determinant for the choice of the animal model in this study. Since we wanted to ensure long-term systemic and local bacterial stimuli, we sensitised the animals with OVA and LPS and challenged them intranasally with OVA, LPS, and SEB. This dual systemic/local focus was critical, as while some researchers reported that the intraperitoneal injection of LPS alone induces neutrophilic inflammation^16^, while others showed that systemic LPS administration inhibits airway inflammation, which is only counteracted by local LPS administration^15^.

Nevertheless, the relevance of murine models in human diseases has been questioned because human conditions cannot be fully modelled or actually develop differently in mice. Inbred mouse strains represent limited genetic diversity and may not reflect the responses generated in genetically polymorphic human populations^41^. Furthermore, the murine model is limited as mice have small sinus cavities and the maxillary sinuses are not completely enclosed by the maxilla^42^. However, murine models are invaluable in vivo models for examining a variety of human diseases that cannot be possible via in vitro experiments using the middle or inferior turbinate mucosa. In addition, rabbit models are sometimes considered superior to mice because rabbits have well pneumatised sinus cavities, and their morphological features are highly similar to that of the human sinonasal epithelium, as opposed to that of mice^43^. However, rabbit models are unsuitable to explore the underlying immunopathological mechanisms related to sinus diseases. In contrast, murine models are generally considered suitable for investigating the sinus disease and have been widely applied for studies understanding the nasal polypogenesis and molecular immune responses in CRSwNP^44^. Most recently, Kim et al. showed that the nasal polyp mouse model demonstrates enhanced B cell responses reminiscent of B cell responses in human nasal polyp^45^.

In conclusion, our study shows that the administration of LPS or poly(I:C), as bacterial- and viral-derived components, respectively, in a murine model of allergic rhinosinusitis with NP formation leads to the development of different inflammatory profiles but does not influence NP formation per se. While LPS induced a predominant Th1/Th17 environment, poly(I:C) contributed towards a Th2-skewed environment. Therefore, our data may have implications in the physiopathology of CRSwNP with a known complex aetiology.

## Methods

### Ethics statement

All animal experiments were performed in compliance with the Seoul National University Animal Care and Use Committee guidelines and approved under the reference code SNU-170203-3-2.

### Experimental animals

Four-week-old BALB/c mice (weighing 20–25 g) were used as the experimental animals. Animals were kept in a special pathogen-free biohazard containment facility maintained at 22–24 °C and 50–60% humidity.

### Experimental groups and sensitization/challenge protocols

**Figure 5:**
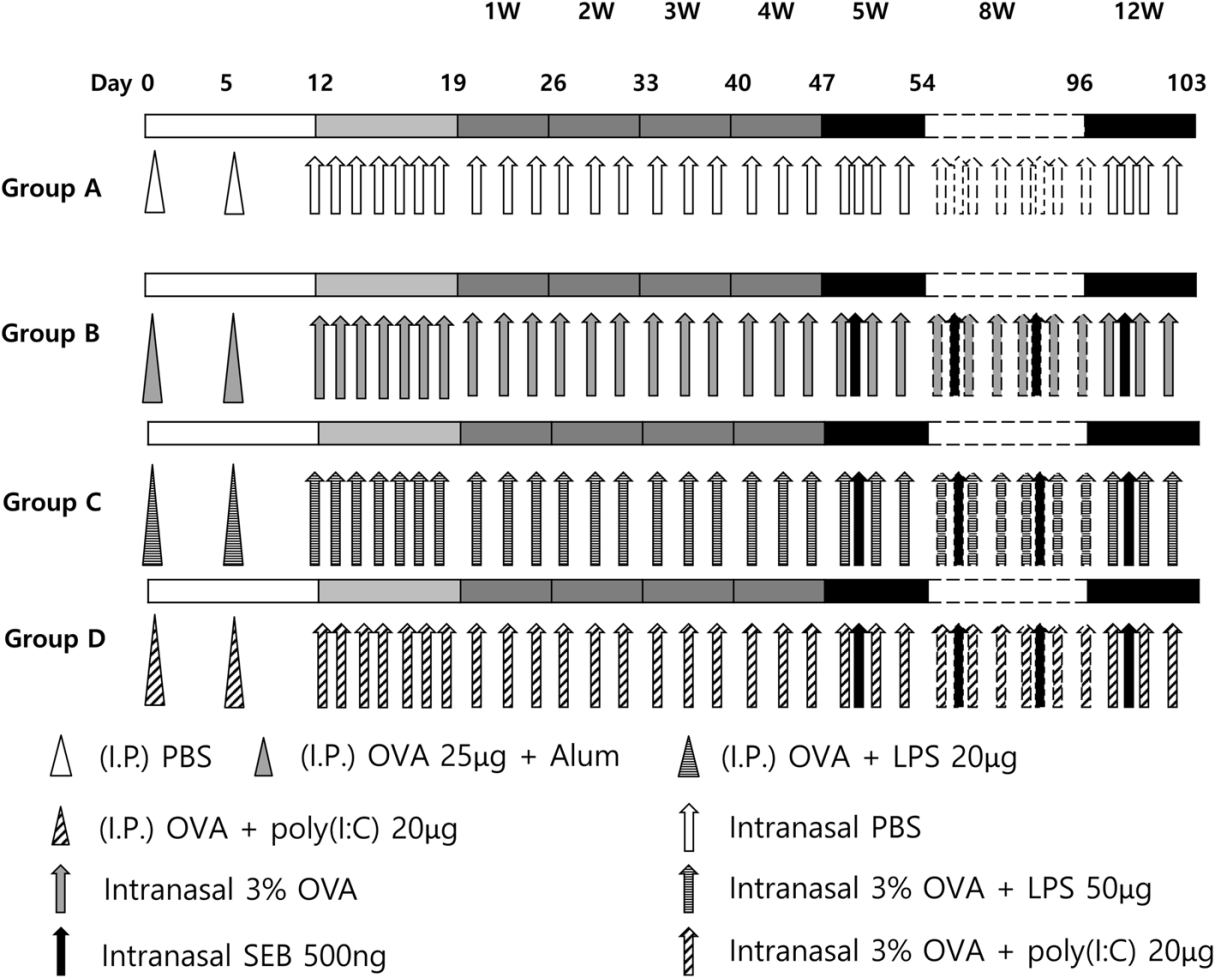
Experimental protocol. This figure was drawn using Microsoft’s PowerPoint 2016 (https://www.microsoft.com).

The general experimental layout is summarised in Fig. 5. Mice were randomly divided into 4 groups having ten mice each: Group A: negative control group, Group B: positive control group, Group C: LPS stimulation, and Group D: poly(I:C) stimulation. Allergic rhinosinusitis was induced in all mice, excluding the ones in the negative control group, following a previously established protocol^8^. Briefly, mice were first sensitised with 25 μg of OVA (grade V; Sigma-Aldrich, St. Louis, MO, USA) in complex with 2 mg of aluminium hydroxide gel adjuvant (alum) (Thermo Fisher Scientific, Rockford, IL, USA) via intraperitoneal injection on days 0 and 5. The mice were then challenged intranasally with 3% OVA diluted in 40 μL of phosphate-buffered saline (PBS) daily, from days 12 to 19, followed by three times a week thereafter for 12 consecutive weeks. Simultaneously, from week 5, considering the triweekly instillations, mice were intranasally challenged on a weekly basis with SEB (500 ng) (Product# 122; List Biological Laboratories, Inc., Campbell, CA, USA). Additionally, LPS (#L2880; Sigma-Aldrich) and poly(I:C) (#P1530; Sigma-Aldrich), were added to the sensitization (20 μg of each) and challenge (50 μg and 20 μg, respectively) mixtures for the experimental groups C and D, respectively. In parallel, PBS was always administered to the negative control animals via the same administration routes.

### Histopathological analysis

For the histopathological analysis, the heads of five mice per group were collected. Detailed experimental procedures have been previously described^46,47^. Different protocols were used to evaluate the degrees of inflammation, polyp formations, and epithelial disruptions. Hematoxylin and Eosin (H&E) staining was used for the observation of tissue architecture and cellularity; Sirius red (Polysciences Inc., Warrington, PA, USA) staining, for eosinophils; anti-neutrophilic antibody (NIMP-R14; Abcam, Cambridge, UK) staining, for neutrophil; Giemsa (Sigma-Aldrich) staining, for mast cells; and Periodic acid‑Schiff (Sigma-Aldrich) staining, for goblet cells.

The numbers of positive cells were determined in five high-power fields (HPF; 400×) by two independent observers who were blinded to the group assignment. If the examiners had a disagreement, a consensus was reached by reviewing the specimen under a multihead microscope by our research team. NPs were defined as distinct mucosal bulges with neutrophilic infiltration and/or microcavity formation, as previously described^8^. The results of inflammatory and secretory cells were expressed as cells per high-power field.

### Cytokine expression analysis

The nasal mucosa of the remaining five mice in each group was carefully taken out using a curette. Total RNA was isolated from the nasal mucosa samples using the TRIzol reagent (Invitrogen, Carlsbad, CA, USA). Complementary DNAs (cDNAs) were synthesised using amfiRivert Platinum cDNA Synthesis Master Mix (GenDEPOT, Katy, TX, USA). For the analysis of (IL)-4 (Mm 00445259_m1), IL-5 (Mm 00439646_m1), IL-13 (Mm00434204_m1), IFN-γ (Mm99999071_m1), IL-17A (Mm00439618_m1), IL-23 (Mm00518984_m1), MMP9 (Mm00442991_m1), IL-25 (Mm00499822_m1), IL-33 (Mm00505403_m1), TSLP (Mm00498739_m1), and glyceraldehyde-3-phosphate dehydrogenase (GAPDH) (Mm99999915_g1), predeveloped assay reagent kits of primers and probes were purchased from Applied Biosystems (Foster City, CA, USA). Amplification of cDNAs was performed in MicroAmp optical 96-well reaction plates (Applied Biosystems) with TaqMan Universal PCR Master Mix (PE Biosystems, Foster City, CA, USA), using an ABI PRISM 7000 Sequence Detection System (Applied Biosystems, Foster City, CA, USA). GAPDH was used as an endogenous control.

### Quantification of serum total and OVA-specific IgE levels

Serum samples from mice were obtained at the time of sacrifice. Serum total and OVA-specific IgE levels were measured by enzyme-linked immunosorbent assay (ELISA) as described previously^48^. Briefly, for the analysis of total IgE, serum samples were added to the 96-well plates along with purified mouse IgE isotype (#557079, BD Biosciences) used as a standard. For the analysis of OVA-specific IgE, serum samples were added to the OVA (100 μg/mL in 0.05 M carbonate-bicarbonate buffer)-coated plates 96-well flat-bottom plates. After following the outlined protocols, plates were then washed three times and developed with 100 μL per well of 3,3′,5,5′-tetramethylbenzidine (TMB) (#52-00-00, KPL). The reaction was terminated by the addition of 1 N HCL (50 µl/well). Optical density was measured in a microplate reader at 450 nm. Total IgE levels were determined by interpolation from a standard curve.

### Statistical analyses

The data are represented as means ± SEM. Illustrative figures were generated using Prism version 8.0 (GraphPad Software Inc., La Jolla, CA, USA). Statistical analysis was performed using SPSS 24.0 software (IBM, Armonk, NY, USA). Mann–Whitney U and Kruskal–Wallis tests were used to compare differences between two, or more than two groups, respectively. Statistical significance was given by a P value inferior to 0.05.

## Data Availability

All relevant data are within the paper and available from the corresponding author on reasonable request.
